# Fostering Socially Accountable Physicians: Medical Students’ Perspectives on the Educational Impact of a Longitudinal Service-Learning Course Series

**DOI:** 10.7759/cureus.106658

**Published:** 2026-04-08

**Authors:** Lizzeth N Alarcon, Rachel D Clarke, Onelia Lage, Nana Garba

**Affiliations:** 1 Department of Medical Education, Florida International University Herbert Wertheim College of Medicine, Miami, USA

**Keywords:** interprofessional education, longitudinal curricula, medical education, service-learning, social accountability

## Abstract

Background

There is a growing consensus that longitudinal service-learning curricula can be more effective in promoting social accountability than shorter-term elective experiences. The Florida International University Herbert Wertheim College of Medicine (FIU HWCOM) has a required longitudinal, service-learning course series, the Community Engaged Physician (CEP), where students conduct household visits in underserved Miami-Dade County communities through the Green Family Foundation’s Neighborhood Health Education Learning Program (NeighborhoodHELP).

Aim

This cross-sectional study examined FIU HWCOM’s graduating medical students’ perspectives on the perceived impact that CEP/NeighborhoodHELP has had on their medical education.

Methods

A Qualtrics survey (Qualtrics, Provo, UT, USA) was developed using the courses’ learning objectives and validated with the third-year students (class of 2025) at our medical school. A week before graduation, the validated survey was shared with the class of 2024. The survey included specialty choice and five-point Likert-type questions assessing the perceived impact of CEP/NeighborhoodHELP on students’ education in the areas of interprofessional teamwork, the social determinants of health (SDOH), and care of diverse populations. Descriptive analyses were conducted to assess student responses.

Results

Fifty-one students completed the survey, resulting in a response rate of 53% among those present (51/96), which represents 42.5% of the graduating class (51/120). Most respondents (68%) were satisfied with their CEP/NeighborhoodHELP experience. Eighty-eight percent reported increased confidence in their interprofessional teamwork and a stronger understanding of physicians’ roles in promoting health equity.

Conclusion

Our graduating students perceived the structured service-learning experiences in CEP as impactful. Integrating service-learning as a required and longitudinal component of the curriculum yields highly positive and meaningful student experiences.

## Introduction

Over the past few years, health policy researchers have emphasized that a population health approach is needed to improve health outcomes [1]. Social determinants of health (SDOH), including poverty, unsafe living conditions, and limited healthcare access, are interrelated and significantly contribute to health disparities [2]. As such, medical education has had a renewed emphasis on recognizing and addressing the SDOH with the goal of preparing future health professionals to better care for underserved communities [3]. The Liaison Committee on Medical Education (LCME) includes standards that guide curricular content for medical schools in the United States; under Standard 7, elements 7.5 and 7.6 are specifically related to students learning about societal problems, healthcare disparities, and provision of care to a diverse society [4].

Service-learning has traditionally been a way to expose medical students to underserved communities. It combines the goals of community service with student learning objectives to enhance the learning and service experience [5,6]. It is also associated with more favorable views among medical students regarding working with communities that experience greater SDOH needs, fostering empathy and social responsibility [7-9]. Moreover, having community health field experiences and cultural humility training has been found to be statistically associated with positive changes in medical students' intention to practice in underserved areas [10]. Service-learning experiences also enhance students’ leadership skills and prepare them for future roles in patient advocacy, research, and policy development [11,12]. However, these experiences are often structured as electives or as extracurricular activities with variable longitudinal components [13-15]. There is growing consensus that a structured service-learning curriculum that is greater in length and combines educational methods can be more effective in promoting social accountability [16, 17]. A scoping review published in 2023 assessing the effectiveness of integration of SDOH curricula into medical education noted variable lengths in programming ranging between three and 18 months [16]. The literature on the integration of SDOH curricula and service learning throughout the four years of medical school, as well as related educational outcomes, is limited.

In line with its mission to train socially accountable physicians, the Florida International University Herbert Wertheim College of Medicine's (FIU HWCOM) Community Engaged Physician (CEP) Course series provides a required, structured, longitudinal curriculum that emphasizes SDOH, cultural humility, and interprofessional education [3]. Students begin the course series during their first year of medical school and continue until graduation. During the first two academic years, they attend twice-monthly didactic sessions that are grounded in systems thinking and community-based engagement. The two-hour classroom experience uses a combination of large-group lectures, small- and large-group activities, case-based discussion, and reflective teaching methods. Experiential learning comes from the service-learning component that students initiate at the end of their first-year course. Students conduct faculty-supervised interprofessional household visits with uninsured and underinsured household members in Miami-Dade County through the Green Family Neighborhood Health Education and Learning Program (NeighborhoodHELP). Medical students are assigned a specific household that they follow from the end of their first year through their fourth year of medical school, with communication check-ins every two months and a total of nine household visits. They conduct telehealth and in-person visits along with students from other health professions, including nursing, physician assistant, and social work students. During the one-hour-long interprofessional household visits, students perform health and social needs assessments and address identified household needs by navigating household members to community-based social services and providing health education, such as nutritional guidance and preventive health screenings [3, 18]. There are formative assessments throughout the course series related to students’ communication skills, teamwork, knowledge of health-related social needs, professional behaviors, and reflective practice. The course series is graded pass or fail.

Given the limited literature on longitudinal service-learning course series and their related outcomes, this study aimed to examine graduating students’ perspectives on the impact that this integrated, four-year curricular experience had on their medical training and education. We used a survey instrument to examine students’ self-reported perceptions and confidence in the areas of interprofessional teamwork, understanding of SDOH, and care of diverse and underserved populations. We secondarily assessed the students’ perceptions of the impact the course series had on their professional development as it relates to residency preparation.

This article was previously presented as an oral presentation at the Association of American Medical College's (AAMC) Southern Group on Educational Affairs Joint Regional Conference in Miami, Florida, on April 29, 2025.

## Materials and methods

Survey development

A Qualtrics survey (Qualtrics, Provo, UT, USA) was developed by a group of content experts to assess students’ experience in CEP/NeighborhoodHELP using course objectives as a guide to formulate the questions. The survey was administered to a group of third-year medical students (class of 2025) to assess the instrument's face validity since they were most representative of the ones who would be taking the survey in a few months. Eight students participated in the face validation process. After taking the survey, students participated in a focus group where they provided feedback on length, clarity, ease of completion, suitability for students participating in the course series, and whether any pertinent topics were missing. Student feedback was reviewed and applied before the survey was administered to the graduating class. The survey consisted of 15 questions and collected basic information about students’ graduating class and specialty of choice. Students were asked to what extent the NeighborhoodHELP program influenced their decision to choose FIU HWCOM with a four-point Likert scale response option ranging from “to a great extent," "to some extent," "to a very little extent," and "not at all.” The following nine questions used a five-point Likert scale from "strongly agree" to "strongly disagree" to assess students’ perceptions of the influence CEP/NeighborhoodHELP had on their choice of specialty, confidence in their ability to work effectively in an interprofessional team, empathy with diverse patient populations, compassion for diverse patient populations, comfort using interpreter/language services, awareness or knowledge of social barriers to improving overall health, ability to develop a clinical and social assessment and plan for patients, and understanding the role physicians play in promoting health equity, as well as the value added to residency applications and interviews. There were also questions about the influence of the CEP/NeighborhoodHELP experience on research interests, overall satisfaction with their CEP experience, and an open-ended question for any additional comments and feedback. The full survey instrument is included in Appendix A.

Survey administration

A QR code to the Qualtrics survey was shared with FIU HWCOM’s graduating class of 2024 during the last week of their final in-house rotation, called Professional Capstone. The survey was administered during this mandatory session to maximize reach; therefore, students absent from this single session (n=24) were unable to participate. Participation was completely voluntary. All students who were members of the graduating fourth-year class at FIU HWCOM and were present for the session to receive the survey link were eligible to participate. There were no exclusion criteria.

Analysis

Descriptive analyses, including univariate analyses, were conducted to assess students’ responses. All analyses were conducted using IBM SPSS Statistics software version 28 (IBM Corp., Armonk, NY, USA). This study was reviewed by the FIU Office of Research Integrity and was deemed exempt via the Exempt Review Process (protocol exemption #IRB-24-0138).

## Results

**Figure 1:**
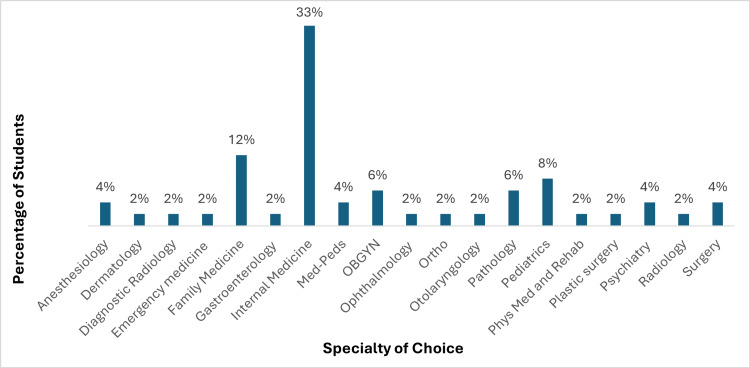
Specialty choice of graduating fourth-year students at FIU HWCOM who completed the survey FIU HWCOM: Florida International University Herbert Wertheim College of Medicine; OBGYN: obstetrics and gynaecology

A total of 120 students were in the graduating fourth-year class. Of these, 96 students were present during the survey session. Fifty-one students completed the survey, yielding a response rate of 53% among those students present (51/96), which represents 42.5% of the total graduating class (51/120). Approximately half (57%) of respondents were pursuing a career in the fields of internal medicine, pediatrics, medicine-pediatrics, obstetrics and gynecology, and family medicine, which are considered primary care. Other specialty choices are noted in Figure 1.

Overall, 68% of respondents indicated that they were either very satisfied or satisfied with their CEP/NeighborhoodHELP experience. Table 1 shows the responses to Likert-type agreement questions about the perceived impact of their experience in CEP/NeighborhoodHELP on their educational training.

**Table 1:** Participant responses to Likert-type agreement questions related to the perceived impact of their experience in CEP/NeighborhoodHELP CEP: Community Engaged Physician; NeighborhoodHELP: Neighborhood Health Education and Learning Program

Question	Strongly Agree, N (%)	Agree, N (%)	Neither Agree Nor Disagree, N (%)	Disagree, N (%)	Strongly Disagree, N (%)
My CEP/ NeighborhoodHELP experience influenced my choice of specialty	1 (2%)	17 (33%)	12 (24%)	12 (24%)	9 (18%)
After my experience with CEP/ NeighborhoodHELP, I am more confident in my ability to work effectively in an interprofessional team.	22 (43%)	23 (45%)	5 (10%)	1 (2%)	0 (0%)
My CEP/ NeighborhoodHELP experience increased my empathy with diverse patient populations. (1 (2%) missing)	20 (39%)	23 (45%)	7 (14%)	0 (0%)	0 (0%)
My CEP/ NeighborhoodHELP experience helped me to have greater compassion for diverse patient populations.	22 (43%)	20 (39%)	8 (16%)	1 (2%)	0 (0%)
My experience with CEP/ NeighborhoodHELP has increased my comfort level when using interpreter/ language services.	17 (33%)	14 (27%)	13 (25%)	4 (8%)	3 (6%)
My experience with CEP/ NeighborhoodHELP has increased my awareness or knowledge of social barriers to improving overall health.	29 (57%)	18 (35%)	4 (8%)	0 (0%)	0 (0%)
My experience with CEP/ NeighborhoodHELP has improved my ability to develop a clinical and social assessment and plan for patients.	20 (39%)	22 (43%)	6 (12%)	3 (6%)	0 (0%)
My experience with CEP/ NeighborhoodHELP has strengthened my understanding of the role physicians play in promoting health equity. (1 (2%) missing)	21 (41%)	24 (47%)	4 (8%)	1 (2%)	0 (0%)
My CEP/ NeighborhoodHELP experience added value to my residency applications and interviews.	27 (53%)	19 (37%)	3 (6%)	2 (4%)	0 (0%)

In the areas related to working with underserved communities, 84% of survey respondents either strongly agreed or agreed that their experience in CEP/Neighborhood HELP increased their empathy, and 82% reported increased compassion when caring for these communities. Similarly, 88% of respondents either strongly agreed or agreed that their service-learning experience strengthened their understanding of the role physicians play in promoting health equity, while 92% of respondents strongly agreed or agreed that they had increased awareness or knowledge related to social barriers’ role in improving overall health.

In regard to students’ perceived clinical competencies, 82% of respondents strongly agreed or agreed that their CEP/NeighborhoodHELP experience improved their ability to develop clinical and social assessments and plans. Moreover, 88% of survey respondents either strongly agreed or agreed that, after their experience with CEP, they were more confident in their ability to work effectively in an interprofessional team.

**Figure 2:**
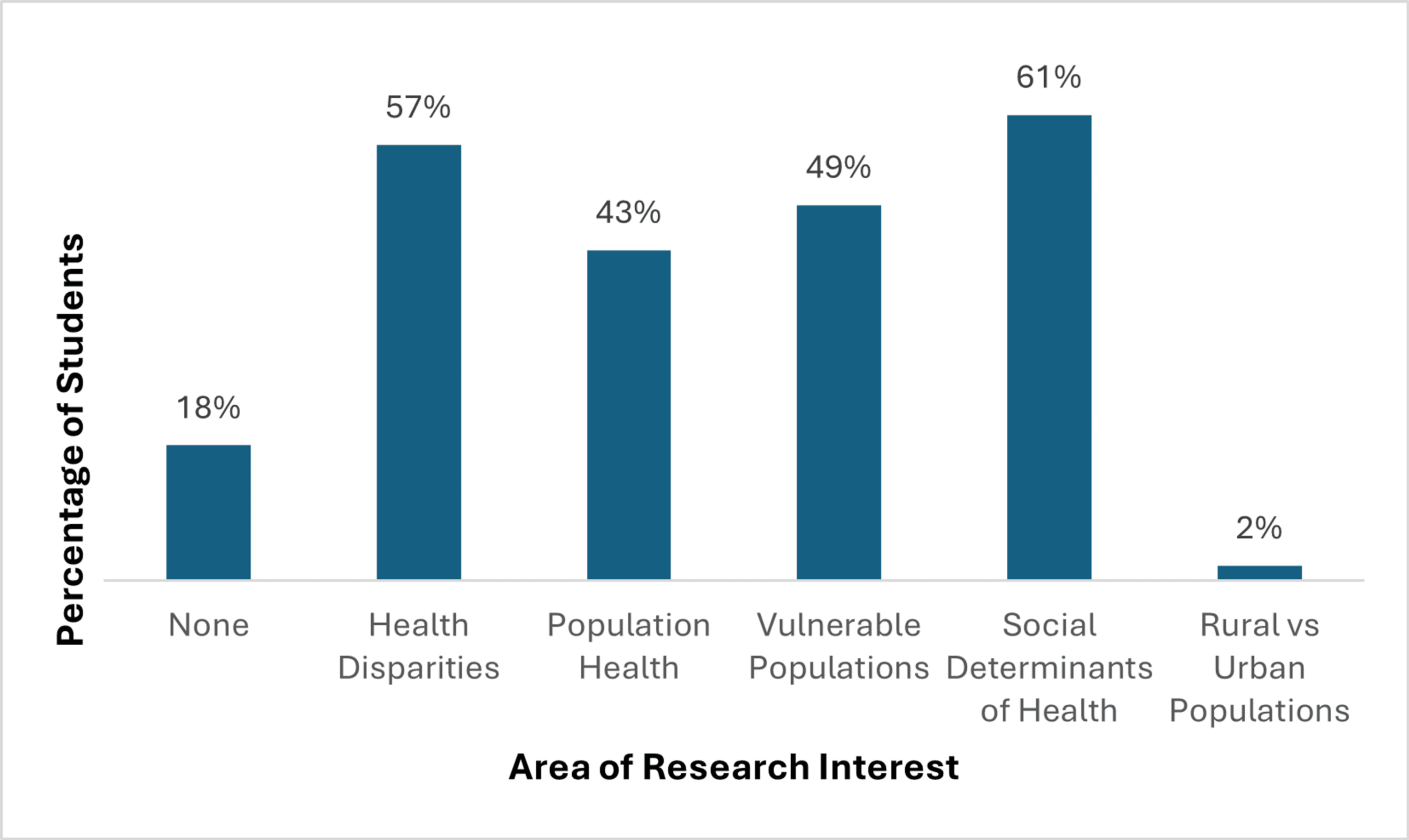
Research topics of interest for survey respondents based on their CEP/NeighborhoodHELP experience CEP: Community Engaged Physician; NeighborhoodHELP: Neighborhood Health Education Learning Program

In terms of perceived impact in scholarly pursuits and preparation for residency applications, a majority of respondents (82%) noted that their experience with CEP/NeighborhoodHELP led them to develop an interest in research in the areas of health disparities, population health, and vulnerable populations, as shown in Figure 2. Furthermore, 35% of respondents either strongly agreed or agreed that their CEP/NeighborhoodHELP experience influenced their choice of specialty, while 90% of respondents strongly agreed or agreed that this experience added value to their residency applications and interviews.

## Discussion

Overall, the graduating medical students at FIU HWCOM who responded to this survey reported a positive experience of their longitudinal, curricular, service-learning program. Most survey respondents agreed that CEP/NeighborhoodHELP had notably impacted their training in all the areas assessed, including addressing the social determinants of health and caring for diverse patients. These findings are consistent with prior literature documenting the importance of incorporating service-learning experiences and working with underserved communities as part of medical training [5-10]. Survey respondents also reported increased empathy and compassion when working with communities, as well as increased awareness of how social barriers impact health. Additionally, survey respondents reported that this service-learning experience, which allows students to work together with students from other health professions, increased their confidence in their ability to work effectively in interprofessional teams. Our study also confirms the growing consensus that structured and longitudinal experiences are impactful [16, 17, 19]. We believe that the fact that CEP was integrated into the curriculum as a structured, required, longitudinal course contributed to the highly positive responses. Continued learning throughout the students’ four years of medical school allows for ongoing exposure to underserved communities and generates meaningful experiences that students value. Other institutions have chosen to implement similar longitudinal curricula with a service-learning component, specifically in their health system science courses [20, 21]. For example, the Keck School of Medicine at the University of Southern California also delivers a longitudinal, required course, Health Justice and Systems of Care, that spans pre-clerkship and clerkship phases of the curriculum and includes a service-learning component [20]. Preliminary evaluations of these curricula show positive reviews from students, though outcomes related to the impact of the service-learning component are not yet available. CEP incorporates elements of the health system science and community-oriented primary care frameworks, with experiential learning that allows the students to apply what they have learned about social barriers to care, interprofessional teamwork, cultural humility, and leveraging community resources in Miami.

Our participants reported that their CEP/NeighborhoodHELP increased their knowledge of social barriers to health and their ability to develop a clinical and social assessment and plan for patients. Regarding working with interpreters, only 60% of respondents agreed that their CEP experience had increased their comfort level when working with interpreters. This is lower than agreement levels for other skill areas. Although we did not ask about the respondents’ specific language skills, this lower agreement frequency could be due to language concordance between our students and their assigned household members. Many of our household members are proficient in English; in addition, many of our students and faculty speak Spanish as a second language, the predominant non-English language spoken by NeighborhoodHELP households in Miami-Dade County.

Furthermore, the graduating class felt their experience strengthened their professional development as they chose their specialty and completed the residency application process. This highlights the potential impact that service-learning experiences and working with vulnerable populations can have on the larger career trajectory of a medical professional in the early stages of their career. Other institutions have also found a similar relationship with professional identity formation and appreciation of physicians’ role in the community and advocacy [19, 22]. For schools prioritizing community engagement and social accountability in their training, our program highlights the importance of making service-learning a curricular priority. Training socially accountable physicians is imperative to addressing health inequities and the needs of vulnerable communities. Our survey did not ask specific questions to characterize how this experience specifically influenced the respondents’ specialty choice compared to their plan when entering medical school. This is an area to further explore in future studies.

Our study is not without limitations. Our survey response rate was modest at 53% at a single recruitment session (42.5% of the total class), introducing non-response and selection bias. Students who completed the survey may have had a more positive experience than those who chose not to participate. Moreover, the students (20% of the graduating class) who were absent during the session when the survey was administered may have had very different perceptions about the course series. Additionally, having baseline data for the study participants prior to their participation in the CEP course series would better inform changes in their confidence regarding interprofessional teamwork, the social determinants of health, and care of diverse populations over the course of their medical school education. Because all students participate in this course, we do not have a control group at our institution. Comparing our outcomes to those of other institutions with shorter-term or elective experiences could be an area of further study. Self-reported data may also be susceptible to social desirability bias, particularly in a required curriculum emphasizing community engagement. Due to the nature of this cross-sectional study, we cannot infer causality and instead present the observed perceptions from the graduating students surveyed.

Furthermore, the outcomes we present here rely on student perceptions about their knowledge and confidence in assessing SDOH, working with interprofessional teams, and diverse populations, which equate to level 1 on the Kirkpatrick Model of learning evaluation [23]. Behavioral change and patient outcomes were not assessed. Examining the completed rubrics with faculty observations of the students’ behaviors in these domains and gathering household-specific outcomes would provide a higher-level evaluation of our curricula in the future. Additionally, although the in-class curriculum is the same for all students as they advance through the CEP course series, there is some variability in the service-learning experience based on household assignments. There are some students who have the same household throughout their time at FIU HWCOM, while other students must get new household assignments when community members leave the program. This can largely influence the student experience with CEP, and our survey did not separate questions based on these two components to get a more nuanced perspective. During future surveys, we aim to ask students whether they had multiple households and if they needed to use an interpreter, so we can assess how these factors influenced their experience. The survey instrument we utilized was developed using our specific course learning objectives, and although we incorporated face validity in our approach, our survey instrument is not psychometrically validated, which limits its future applications and generalizability. Other educators seeking to evaluate their programs may need to use other survey instruments. Additionally, there are no Cronbach’s alpha values available for key domains since domains were not assessed by multiple questions. We also acknowledge that since this was an exploratory, descriptive evaluation, Likert‑scale responses were summarized as agreement percentages without reporting medians, interquartile ranges, or inferential statistics, which may limit the granularity and statistical robustness of the findings.

Finally, CEP has been a curricular experience since the founding of HWCOM, and there have been built-in resources to support the longitudinal integration into the curriculum. Other schools seeking to adopt a structured and required service-learning experience must consider the time, administrative, and financial costs needed to sustain a long-term service-learning experience for all students and develop a scalable institutional commitment.

## Conclusions

An integrated, required, longitudinal service-learning experience across the four years of a medical school’s curriculum was perceived as impactful by graduating medical students. The students reported confidence in their training in the areas of interprofessional education, the social determinants of health, and working with vulnerable communities. Despite limitations related to non-response and selection bias and a single-institution context, the high proportions of students reporting improved confidence in interprofessional work and heightened awareness of social barriers highlight the importance of sustained, required service-learning experiences. Our evaluation shows that our program’s longitudinal design contributed to students' self-reported perceptions of having developed meaningful relationships with community members and interdisciplinary teams, as well as fostering their values of empathy, cultural humility, and patient advocacy.

Future research should extend to multi-site comparisons, linking objective evaluations of student competencies to student confidence and long-term tracking of graduates’ practice patterns to better understand the long-term benefits of these training experiences.

## Appendices

Appendix A

Please state your graduating class. Class of ____

What specialty will you be going into? ____________________

1.    To what extent did the CEP/NeighborhoodHELP program influence your decision to choose FIU HWCOM?

To a great extent

To some extent

To a very little extent

Not at All

For questions 2 through 12, please select the degree to which you agree or disagree with each statement.

2.    My CEP/NeighborhoodHELP experience influenced my choice of specialty.

Strongly Agree

Agree

Neither Agree nor Disagree

Disagree

Strongly Disagree

3.    After my experience with CEP/NeighborhoodHELP, I am more confident in my ability to work effectively in an interprofessional team.

Strongly Agree

Agree

Neither Agree nor Disagree

Disagree

Strongly Disagree

4.    My CEP/NeighborhoodHELP experience increased my empathy with diverse patient populations.

Strongly Agree

Agree

Neither Agree nor Disagree

Disagree

Strongly Disagree

5.    My CEP/NeighborhoodHELP experience helped me to have greater compassion for diverse patient populations.

Strongly Agree

Agree

Neither Agree nor Disagree

Disagree

Strongly Disagree

6.    My experience with CEP/NeighborhoodHELP has increased my comfort level when using interpreter/ language services.

Strongly Agree

Agree

Neither Agree nor Disagree

Disagree

Strongly Disagree

7.    My experience with CEP/NeighborhoodHELP has increased my awareness or knowledge of social barriers to improving overall health.

Strongly Agree

Agree

Neither Agree nor Disagree

Disagree

Strongly Disagree

8.    My experience with CEP/NeighborhoodHELP has improved my ability to develop a clinical and social assessment and plan for patients. 

Strongly Agree

Agree

Neither Agree nor Disagree

Disagree

Strongly Disagree

9.    My experience with CEP/NeighborhoodHELP has strengthened my understanding of the role physicians play in promoting health equity.

Strongly Agree

Agree

Neither Agree nor Disagree

Disagree

Strongly Disagree

10.  My CEP/NeighborhoodHELP experience added value to my residency applications and interviews.

Strongly Agree

Agree

Neither Agree nor Disagree

Disagree

Strongly Disagree

11.  My experience with CEP/NeighborhoodHELP has led me to develop an interest in research that focuses on the following: Select all that apply.

A) Social determinants of health

B) Health disparities

C) Population health

D) Vulnerable populations

E) Other __________________

12.  Overall, how satisfied were you with your CEP/NeighborhoodHELP experience?

Very Satisfied

Satisfied

Neither Satisfied nor Dissatisfied

Dissatisfied

Very Dissatisfied

13. Please provide any additional comments/feedback about your CEP/NeighborhoodHELP experience and the impact it has had on your training.
